# Selective inhibitors of nuclear export (SINE)– a novel class of anti-cancer agents

**DOI:** 10.1186/s13045-014-0078-0

**Published:** 2014-10-15

**Authors:** Kaushal Parikh, Shundong Cang, Arunabh Sekhri, Delong Liu

**Affiliations:** Department of Medicine, New York Medical College and Westchester Medical Center, Valhalla, 10595 NY USA; Department of Oncology, People’s Hospital of Henan Province, Zhengzhou, China; Henan Tumor Hospital, Zhengzhou University, Zhengzhou, China

**Keywords:** SINE, KPT-330, Selinexor, Nuclear export

## Abstract

Dysregulation of the nucleo-cytoplasmic transport of proteins plays an important role in carcinogenesis. The nuclear export of proteins depends on the activity of transport proteins, exportins. Exportins belong to the karyopherin β superfamily. Exportin-1 (XPO1), also known as chromosomal region maintenance 1 (CRM1), mediates transport of around 220 proteins. In this review, we summarized the development of a new class of antitumor drugs, collectively known as selective inhibitors of nuclear export (SINE). KPT-330 (selinexor) as an oral agent is showing activities in early clinical trials in both solid tumors and hematological malignancies.

**Electronic supplementary material:**

The online version of this article (doi:10.1186/s13045-014-0078-0) contains supplementary material, which is available to authorized users.

## Introduction

The nucleo-cytoplasmic transport of proteins plays an important role in maintaining normal cellular functions. The nuclear export of proteins depends on the activity of transport proteins, exportins. Exportin-1 (XPO1), also known as chromosomal region maintenance 1 (CRM1), mediates transport of around 220 proteins [1]-[4]. XPO1 is the sole nuclear exporter of several tumor suppressor (TSP), growth regulatory (GRP) proteins. These include p53, p21, p73, Rb1, apc, bcr-abl, FOXO and STAT3. Under physiological conditions, the export of these proteins prevents them from overacting in the absence of DNA injury or other oncogenic activities [5],[6]. In cancerous cells, however, this export of proteins inhibits their tumor suppressor activity and promotes tumorigenesis [6],[7]. Many hematologic and solid tumor malignancies have elevated XPO1 levels [8]-[12]. Therefore, inhibiting XPO1 can be a potential treatment option. In this review, we will discuss a new class of potential antitumor drugs, collectively known as selective inhibitors of nuclear export (SINE). These agents can block the export of TSPs and GRPs, thus maintaining their intranuclear concentration and exert anti-cancer activity.

### Nucleo-cytoplasmic transport and its inhibition by SINE compounds

**Figure 1:**
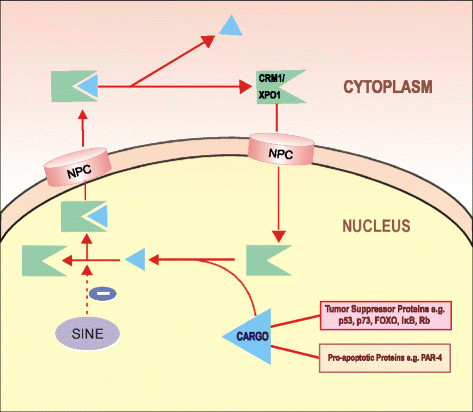
**Mechanism of action for selective inhibitors of nuclear transportation.** In the nucleus, RanGTP and cargo (tumor suppressors, pro-apoptotic proteins, etc.) form a complex with CRM1/XPO1. This complex is exported through the nuclear pore complex (NPC) to the cytoplasm. CRM1 is then recycled back to the nucleus for another round of export. Selective inhibitors of nuclear export (SINE) binds to CRM1/XPO1 and blocks the protein export, and therefore the cargo proteins are retained in the nucleus, leading to growth inhibition.

XPO1 binds to the cargo proteins through a leucine rich nuclear export signal (NES) and transports the proteins through a membrane pore complex via a Ran-GTP gradient [13]-[15] (Figure 1). Several small molecule inhibitors of XPO1 are being studied. These include Leptomycin B (LMB), ratjadone, goniothalamin, N-azolylacrylates, anguinomycin, and CBS9106 [16]-[21]. They bind covalently to the cysteine residue (Cys528) in the NES binding groove of XPO1 [18]. This binding irreversibly inactivates XPO1, leading to intranuclear accumulation of TSPs and GRPs. Of these, leptomycin B (LMB) has been studied most extensively in various cancer cell lines and murine xenograft tumor models.

A phase 1 study of an XPO1 inhibitor showed only modest efficacy and severe dose limiting toxicity (e.g. malaise, anorexia, vomiting and nausea) [22]. The clinical trial was therefore discontinued. KOS-2462, a semisynthetic LMB derivative showed activity in mouse xenograft models without inducing significant toxicity [16]. CBS9106 is another small molecule oral reversible inhibitor of XPO1. It induced growth inhibition in several cancer cell lines [17]. Neither KOS-2462, nor CBS9106 have entered clinical trials.

Subsequently, several novel inhibitors of XPO1, collectively known as SINE compounds, have been developed. These compounds include KPT-330 (selinexor), KPT-335 (verdinexor), KPT-185, KPT-276, and KPT-251. Of these, KPT-185 is the most studied compound *in* vitro with most potency. However, its use *in vivo* is limited by poor pharmacokinetics [23]-[25]. KPT-330 is nearly as potent as KPT-185 and has acceptable oral bioavailability. KPT-251 and KPT-176 are not as potent as KPT-185 but are bioavailable orally [23]-[25]. These agents are currently undergoing clinical trials for several solid and hematologic malignancies including breast, colon, pancreas, renal, multiple myeloma (MM), mantle cell leukemia (MCL), chronic lymphocytic leukemia (CLL), and acute myeloid leukemia (AML).

## SINE in pancreatic cancer

KPT-127, KPT-185, KPT- 205, and KPT-227 were studied in pancreatic cancer cell lines [26]. Prostate apoptosis response-4 (PAR-4) is a proapoptotic protein in the nuclear and cytoplasmic compartments. PAR-4 translocates to the nucleus via XPO1 in external stress conditions to cause apoptosis [27]. PAR-4 is downregulated in pancreatic cancers. Downregulation of PAR-4 directly correlates to worsening outcomes in pancreatic cancer [28]. KPT-185 was shown to increase intranuclear PAR-4 without interfering with its import from the cytoplasm. It also induced PAR-4 phosphorylation, thus activating it and leading to apoptosis. Active SINEs had a median inhibitory concentration (IC_50_) of 150 nmol/L and inhibited pancreatic cancer cell lines while sparing normal human pancreatic ductal epithelial cells. The *in vivo* effects were noted using KPT-330 (selinexor) in subcutaneous and orthoptic pancreatic cancer models in mice. Oral administration of KPT-330 led to significant tumor growth inhibition when compared with control or gemcitabine treatment [26]. KPT-330 treated mice had drastic reductions in tumor size as compared with controls. Thus, pre-clinical studies of CRM1 inhibition using SINE compounds revealed an attractive novel treatment of pancreatic cancer.

## SINE in triple-negative breast cancer (TNBC) (ER−, PR−, Her2−)

Overexpression of survivin is associated with poor prognosis in breast cancer [29]. Survivin inhibits apoptosis by stabilizing X linked inhibitor of apoptosis (XIAP) in the cytoplasm [30]. Survivin expression is also directly affected by STAT3, a member of Janus-activated kinase (JAK)/STAT [31], which is increased in several malignancies including TNBC [32]. Cytoplasmic localization is required for survivin to inhibit apoptosis [30]. XPO1 mediates transport of survivin and STAT3 to the cytoplasm, and inhibits apoptosis [33],[34]. Inhibition of XPO1 blocked STAT3 binding to survivin promoter and decreased survivin expression. In the meanwhile, it was shown that survivin was cleaved by caspase-3, therefore leading to overall decrease of survivin level [4]. In the study, it was shown that KPT-185, KPT-251 and KPT-276 inhibited tumor cell growth and enhanced apoptosis *in vitro* in 3 different cell lines. KPT-185cis had the lowest IC_50_. KPT-330 had profound effects on tumor cell growth inhibition and apoptosis with an IC_50_ ranging from 5 to 21 nmol/L. The data suggested that twice weekly dosing of KPT-330 at 25 mg/kg for 42 days significantly reduced tumor growth when compared to control or standard treatment with 5-fluorouracil (P = 0.011). It was determined that XPO1 inhibition caused nuclear retention of survivin which was then degraded by caspase-3 [4]. Survivin transcription was also shown to be repressed by inhibition of CREB binding protein (CBP) mediated STAT3 transactivation.

This study expands the role of SINEs in treatment of breast cancer and other solid tumors. KPT-330 is currently undergoing phase I clinical trial in advanced solid tumors [35]. In this early trial, KPT-330 was administered orally for 8–10 doses in a 28-day cycles to 103 patients (59/44 M/F; median age 61 years) across 12 dose levels. Dose limiting toxicites (DLT) (fatigue, dehydration, nausea) were noted. Dosing at 65 mg/m^2^ BIW is ongoing since maximal tolerated dosage (MTD) was not reached yet at the time of the report. There were 87 evaluable patients (pts) for response. Among them, there were 3 PR in colorectal cancer (KRAS mutant), melanoma (BRAFwt) and ovarian adenocarcinoma pts. Stable disease (SD) was seen in 39 pts, with 12 pts lasting over 6 months. All 5 evaluable pts with hormone and chemotherapy refractory prostate cancer (HRPC) achieved SD; Nine of 13 evaluable pts with squamous head and neck cancer had SD diseases. Further evaluations are ongoing.

## SINE in non-small cell lung cancer (NSCLC)

Epidermal growth factor receptor-tyrosine kinase inhibitors (EGFR-TKI) are main treatment for patients with advanced NSCLC with EGFR exon 19 deletion or exon 21 substitution [36],[37]. EGFR overexpression and p53 mutations are associated with poor outcomes in NSCLC [38],[39]. As mentioned earlier, nuclear export of p53 is mediated by XPO1 [40]. Sun *et al.* studied the antitumor activity of KPT-330 against NSCLC *in vitro* and *in vivo*, and concluded that the antitumor activity of KPT-330 against NSCLC was independent of p53 mutational status [41]. The antitumor activity of KPT-330 against NSCLC was likely related to p73. p73 shares structural and functional similarities with p53 and cooperates with p53 to induce apoptosis [42]. In cells with mutant p53, p73 is shown to cause apoptosis via activation of p53-inducible genes [42]. KPT-330 caused dose-dependent growth inhibition of NSCLC with correlating decrease in XPO1 levels [41]. Moreover, KPT-330 can inhibit NSCLC cell growth even in EGFR-TKI resistant cancer cells. Combination of KPT-330 and cisplatin displayed synergistic *in vitro* antiproliferative activity. *In vivo* treatment of mice with a dose of 10 mg/kg, thrice weekly for 4 weeks showed significant tumor growth inhibition with minimal toxicities. Another independent study confirmed the above findings using KPT-185 *in vitro* and its oral clinical equivalent KPT-276 on NSCLC cells *in vivo* using mouse xenografts [43].

## SINE in renal cell carcinoma (RCC)

Despite several approved drugs for metastatic RCC, the progression free survival remains only 1 to 2 years [44]. KPT-185 was studied *in vitro* in RCC cell lines [45]. XPO1 is overexpressed in high grade RCC. KPT-185 and its oral equivalent KPT-251 decreased XPO1 levels. They also compared SINE with sorafinib and found greater inhibition of tumor growth with KPT-251 at a higher dose of 75 mg/kg in mouse xenografts (p = 0.07) without any adverse effects. KPT-185 increased nuclear localization of p53 and its downstream protein p21 to cause cell cycle arrest. Also, cytosol p21 reduction leads to apoptosis. KPT-251 showed increased p53 and p21 nuclear levels *in vivo.* Sorafinib, on the other hand, decreased nuclear and cytoplasmic p21, thereby causing apoptosis [46]. These findings warrant further clinical studies using SINE as a treatment choice in RCC.

## SINE in melanoma

BRAF kinase activation is present in about 50% of melanomas [47]-[52]. Treatment with BRAF and MEK inhibitors has been very successful, but the eventual development of resistance to these kinase inhibitors calls for more agents [51],[53]-[60]. XPO1 expression is found to be increased in metastatic melanoma more than primary melanoma or nevi [61]. Hence, SINE can be a potential treatment for metastatic melanoma. Salas Fragomeni *et al.* conducted *in vitro* and *in vivo* studies in metastatic melanoma using SINE and BRAF inhibitors [62]. They concluded that BRAF inhibition by PLX-432 led to inhibition of cell proliferation in BRAF-mutant cell lines, but BRAF wild-type (WT) melanoma cell lines were relatively resistant [53],[54]. However, SINEs inhibited cell proliferation and caused cytotoxicity across all cell lines, regardless of BRAF status. They also showed synergistic activity between SINE and PLX-432 in BRAF mutant melanoma. Furthermore, SINE and MEK inhibitors were also able to synergize with the three-way treatment (SINE/MEK/BRAF), achieving an even lower IC_50_. SINE also caused G1/S phase cell cycle arrest, another effect synergized with concomitant BRAF inhibition in BRAF mutant cell lines. SINE also increased nuclear p53, retinoblastoma (Rb). An increase in ERK phosphorylation in the nucleus was also noticed. ERK phosphorylation has been linked to increased cellular proliferation and development of chemoresistance [61],[63],[64]. However, this SINE- induced ERK phosphorylation was prevented in the presence of BRAF inhibition, possibly explaining the synergy between these two compounds. *In vivo* mouse model showed complete tumor regression using the combination regimen [62].

## SINE in acute leukemia

SINE compounds have been studied as a novel anticancer strategy in multiple preclinical trials of hematologic malignancies. Higher levels of XPO1 are associated with poor prognosis in acute leukemia [65]. Earlier preclinical work demonstrated that KPT-185 inhibited proliferation and induced G1 phase cell cycle arrest in AML cell lines and primary AML blasts *in vitro*[25]. Nucleophosmin 1 (NPM1) is a nucleolar TSP that shuttles between the nucleolus and cytoplasm via the XPO1-RanGTP pathway and regulates p53 dependent cell death [66]. NPM1 mutations in AML cells were seen in 25% to 35% cases [67]. These mutations cause increased XPO1 binding and localization of NPM1 in the cytoplasm [68]. SINEs block this export of mutant NPM1 and induce antileukemic effects in AML cell lines and primary AML blasts. AML blasts with NPM1 mutations were very responsive to KPT-185 and had an IC_50_ of 100 nmol/L. However, wild type (WT) NPM1 in AML cells were also sensitive to SINEs, indicating that other TSPs like p53 also have a role in the antileukemic effects of SINE. Kojima *et al.* further established that p53 is a major determinant in SINE induced cytotoxicity in AML, independent of NPM1. Mutant p53 samples were less sensitive to KPT-185 [65]. XPO1 inhibition by SINE also resulted in blast differentiation, likely due to upregulation by p53 and CEBPA [69], a protein essential for myeloid granulocytic differentiation via activation of several necessary genes [70],[71]. SINEs were also shown to downregulate FLT3 and cKIT tyrosine kinase proteins [25]. FLT3 gene mutation may coexist with NPM1 mutations [72]. SINE downregulated FLT3 and NPM1. Thus, SINEs can potentially target 2 critical pathways. cKIT mutations or overexpression also confer a worse prognosis in AML [73],[74]. Kojima *et al.* demonstrated synergistic activity using the combination of SINE with MDM2 inhibitor Nutlin-3a. MDM2, frequently overexpressed in AML, is a p53-specific ligase, promoting p53 degradation [75]. Nutlin-3a is a selective MDM2 inhibitor, shown to increase nuclear and cytoplasmic p53 and induces p53 mediated apoptosis [75]. The addition of SINE to Nutlin-3a led to higher p53 nuclear level than by using either agent alone *in vitro*[65]. This combination strategy can be potentially effective not only in AML but in several other malignancies. Furthermore, SINEs are not shown to induce apoptosis in normal hematopoietic cells [23],[65],[76].

T cell-acute lymphoblastic leukemia (T-ALL) is fatal in about 50-70% of adult patients [77]-[79]. SINE has shown striking activity in preclinical study for treatment of T-ALL. KPT-185 and KPT-330 showed rapid apoptosis induction in T-ALL cell lines *in vitro* with acceptable IC_50_[76]. *In vivo* treatment showed minimal gastrointestinal adverse effects.

Philadelphia chromosome positive ALL remains a challenge even with availability of multiple tyrosine kinase inhibitors [80],[81]. Walker *et al.* demonstrated successful use of KPT-185 *in vitro* and KPT-330 *in vivo* in Philadelphia chromosome positive ALL (Ph + ALL) and chronic myeloid leukemia blast crisis (CML-BC). Combination with imatinib led to synergistic effects [82]. SINE treatment was associated with significant reduction in BCR-ABL + cells in mice, likely by reactivation of the tumor suppressor proteins PP2A, p53, p21 and FOXO3a [82].

The preliminary results of an ongoing phase I trial using KPT-330 in relapsed/refractory AML were presented Yee *et al.* recently. They showed that KPT-330 treatment given to heavily pretreated, refractory/relapsed AML patients had no DLT. Out of the 32 evaluable patients, 4 (12%) showed complete response (CR) with hematological recovery, 1 (3%) showed marrow CR (mCR), mCR without hematological recovery was seen in 1 (3%) patient. Partial response (PR) was seen in 2 patients (6%). Eleven patients (34%) showed progression while 12 (37%) experienced stable disease after 30 days [83].

## SINE in chronic leukemia

Even though more options are available now for chronic lymphocytic leukemia (CLL) therapy, p53 positive CLL still has poor prognosis [84]-[91]. Preclinical study by Lapalombella *et al.* using KPT-185 *in vitro* and KPT-251 *in vivo* showed promising results in chronic lymphocytic leukemia (CLL) cells [24]. KPT-185 induced nuclear retention of IκB. IκB is an endogenous inhibitor of the inflammatory antiapoptotic transcription factor NFκB, which is involved in the upregulation of MCL1, the most significant antiapoptotic protein associated with CLL [92],[93]. KPT-185 induced MCL1 depletion, likely due to inactivation of NFκB by nuclear retention of IκB. Murine xenografts treated with KPT-251 showed significant improvement in survival when compared with fludarabine [24]. In a 37 year old patient with CML-AP resistant to multiple treatment options (TKIs, interferon, omacetaxine and azacitidine), a trial of selinexor (KPT-330) on a compassionate use protocol showed significant reduction in bone pain, spleen size, white blood cell count, and lactate dehydrogenase (LDH) level. Peripheral blood smears showed a dramatic reduction in immature myeloid blasts [82].

## SINE in multiple myeloma (MM)

SINE was shown to induce cytotoxicity and inhibits osteoclastogenesis in multiple myeloma *in vitro* and *in vivo*[12]. High CRM1 expression was found to be associated with lytic bone disease (P = 0.008) and shorter survival (P = 0.024). CRM1 levels are higher in bortezomib resistant MM cells. SINEs caused nuclear accumulation of multiple TSPs including p53, FOXO3a, IκB, p21 and PP2A. SINE also caused anti-MM effects in the bone marrow microenvironment by activating caspase cascade and causing PARP cleavage, and showed a synergistic effect when combined with bortezomib, without affecting bone marrow stromal cells. Another study, however, observed no synergy with KPT-276 in combination with dexamethasone, bortezomib, or melphalan [94]. Osteoclastogenesis is controlled by NFκB activation through cytokine RANKL and NFAT1c. Both KTP-185 and KPT-330 blocked RANKL mediated activation of NFκB in osteoclast (OC) precursor cells, and also blocked NKFAT1c, which is also essential for osteoclast function [12],[94]. Surprisingly, SINE reduced the expression of oncogene c-myc, despite the fact that CRM1 does not mediate c-myc export [95]. C-myc activation is associated with poor prognosis and shorter survival in monoclonal gammopathy of undetermined significance (MGUS) and MM [96]. A study done in 2012 indicated that p53 activation is responsible for inhibiting CRM1 and c-myc genes [97]. BRD4, another gene downregulated by SINE, regulates DNA replication, promotes c-myc transcription and is associated with MM disease progression [98],[99]. BRD4 knockdown causes cell cycle arrest and subsequent apoptosis [98]. JQ1, a small molecule inhibitor of BRD4 gene causes decreased transcription of c-myc, an effect that was synergistic with KPT-276 [98]. This result can be utilized for further cytotoxic treatment of tumor cells in MM. Lastly, 2 week *in vivo* treatment with KPT-276 had a comparable effect on M-spike reduction with melphalan and bortezomib, the two potent anti-MM drugs [94].

## SINE in lymphoma

Novel agents and regimens for lymphoma are moving rapidly from bench to bedside [89],[100]-[104]. KPT drugs have been studied preclinically for the treatment of resistant mantle cell lymphoma (MCL). Yoshimura *et al.* conducted an in vitro study of KPT-185 in MCL and showed that SINE increases MCL cell apoptosis primarily by increasing nuclear p53 levels [105]. They verified that KPT-185 downregulated c-myc and NFκB, thus targeting multiple pathways of apoptosis. Zhang *et al.* studied the *in vivo* effects of KPT-276 in mice and showed marked activity with minimal weight loss, gastrointestinal side effects, or myelosuppression [106]. London *et al.* studied SINEs *in vivo* in a phase I clinical trial in spontaneous canine NHL, osterosarcoma or mast cell tumor. They used KPT-335 at a dose of 1 mg/kg to 1.75 mg/kg two times a week and showed significant response without development of serious side effects [107]. Gutierrez *et al.* presented the findings of their phase I study with KPT-330 in 32 pretreated refractory lymphoma patients. The optimal dosing of KPT-330 is at least 45 mg/m^2^ and durable activity of KPT-330 was observed in those NHL patients [108]. These data further validated the activity of SINEs in human malignancies.

## Future directions

More and more targeted small molecule inhibitors are entering clinical application quickly [53],[84],[85],[109]-[113]. Selective inhibitors of nuclear export (SINE) show activity in a wide variety of cancers, both hematologic and solid tumors [114],[115]. Currently, they are being studied in early phase clinical trials (Table 1). Their low toxicity profile and synergistic effects in combination with other antineoplastic agents support further development in combination regimens against a wide range of malignancies. SINEs represent a unique, novel class of targeted agents for various malignancies.

**Table 1:** **Selinexor (KPT-330) in clinical trials**

Diseases	Trial	Recruting status	NCT number
Relapsed ALL and AML	Phase I	Recruiting	NCT02091245
Unresectable melanoma	Phase I	Not yet Recruiting	NCT02120222
Gynaecologic malignancies	Phase II	Recruiting	NCT02025985
Advanced/metastatic solid tumors	Phase I	Recruiting	NCT02078349
Soft-tissue or bone sarcoma	Phase Ib	Recruiting	NCT01896505
Advanced/metastatic solid tumors	Phase I	Recruiting	NCT01607905
Metastatic resistant prostate cancer	Phase II	Recruiting	NCT02146833
Advanced hematological malignancies	Phase I	Recruiting	NCT01607892
Recurrent glioblastoma	Phase II	Recruiting	NCT01986348
Relapsed/refractory AML	Phase II	Rectruiting	NCT02088541
Refractory/relapsed CLL	Phase II	Recruiting	NCT02138786
Acute myeloid leukemia	Phase I	Recruiting	NCT02093403
Locally advanced rectal cancer	Phase I	Not yet Recruiting	NCT02137356

Note: details of all NCT trials can be found on http://www.clinicaltrials.gov.

## Authors’ contributions

DL and KP designed the study. All authors have contributed to data preparation, drafting and revising the manuscripts. All authors have read and approved the final manuscript.

## Authors’ original submitted files for images

Below are the links to the authors’ original submitted files for images. Authors’ original file for figure 1

## References

[1] Fukuda M, Asano S, Nakamura T, Adachi M, Yoshida M, Yanagida M, Nishida E (1997). CRM1 is responsible for intracellular transport mediated by the nuclear export signal. Nature.

[2] Xu D, Grishin NV, Chook YM (2012). NESdb: a database of NES-containing CRM1 cargoes. Mol Biol Cell.

[3] Nguyen KT, Holloway MP, Altura RA (2012). The CRM1 nuclear export protein in normal development and disease. Int J Biochem Mol Biol.

[4] Cheng Y, Holloway MP, Nguyen K, McCauley D, Landesman Y, Kauffman MG, Shacham S, Altura RA (2014). XPO1 (CRM1) inhibition represses STAT3 activation to drive a survivin-dependent oncogenic switch in triple-negative breast cancer. Mol Cancer Ther.

[5] Kutay U, Hetzer MW (2008). Reorganization of the nuclear envelope during open mitosis. Curr Opin Cell Biol.

[6] Turner JG, Sullivan DM (2008). CRM1-mediated nuclear export of proteins and drug resistance in cancer. Curr Med Chem.

[7] Turner JG, Dawson J, Sullivan DM (2012). Nuclear export of proteins and drug resistance in cancer. Biochem Pharmacol.

[8] Noske A, Weichert W, Niesporek S, Röske A, Buckendahl AC, Koch I, Sehouli J, Dietel M, Denkert C (2008). Expression of the nuclear export protein chromosomal region maintenance/exportin 1/Xpo1 is a prognostic factor in human ovarian cancer. Cancer.

[9] Shen A, Wang Y, Zhao Y, Zou L, Sun L, Cheng C (2009). Expression of CRM1 in human gliomas and its significance in p27 expression and clinical prognosis. Neurosurgery.

[10] Yao Y, Dong Y, Lin F, Zhao H, Shen Z, Chen P, Sun YJ, Tang LN, Zheng SE (2009). The expression of CRM1 is associated with prognosis in human osteosarcoma. Oncol Rep.

[11] Huang WY, Yue L, Qiu WS, Wang LW, Zhou XH, Sun YJ (2009). Prognostic value of CRM1 in pancreas cancer. Clin Invest Med.

[12] Tai YT, Landesman Y, Acharya C, Calle Y, Zhong MY, Cea M, Tannenbaum D, Cagnetta A, Reagan M, Munshi AA, Senapedis W, Saint-Martin JR, Kashyap T, Shacham S, Kauffman M, Gu Y, Wu L, Ghobrial I, Zhan F, Kung AL, Schey SA, Richardson P, Munshi NC, Anderson KC (2014). CRM1 inhibition induces tumor cell cytotoxicity and impairs osteoclastogenesis in multiple myeloma: molecular mechanisms and therapeutic implications. Leukemia.

[13] Güttler T, Görlich D (2011). Ran-dependent nuclear export mediators: a structural perspective. EMBO J.

[14] Monecke T, Güttler T, Neumann P, Dickmanns A, Gürlich D, Ficner R (2009). Crystal structure of the nuclear export receptor CRM1 in complex with Snurportin1 and RanGTP. Science.

[15] Fung HY, Chook YM: Atomic basis of CRM1-cargo recognition, release and inhibition.*Semin Cancer Biol* 2014, ., [http://dx.doi.org/10.1016/j.semcancer.2014.03.002] 10.1016/j.semcancer.2014.03.002 PMC4108548 24631835

[16] Mutka SC, Yang WQ, Dong SD, Ward SL, Craig DA, Timmermans PB, Murli S (2009). Identification of nuclear export inhibitors with potent anticancer activity in vivo. Cancer Res.

[17] Sakakibara K, Saito N, Sato T, Suzuki A, Hasegawa Y, Friedman JM, Kufe DW, Vonhoff DD, Iwami T, Kawabe T (2011). CBS9106 is a novel reversible oral CRM1 inhibitor with CRM1 degrading activity. Blood.

[18] Kudo N, Matsumori N, Taoka H, Fujiwara D, Schreiner EP, Wolff B, Yoshida M, Horinouchi S (1999). Leptomycin B inactivates CRM1/exportin 1 by covalent modification at a cysteine residue in the central conserved region. Proc Natl Acad Sci U S A.

[19] Bonazzi S, Eidam O, Güttinger S, Wach JY, Zemp I, Kutay U, Gademann K (2010). Anguinomycins and derivatives: total syntheses, modeling, and biological evaluation of the inhibition of nucleocytoplasmic transport. J Am Chem Soc.

[20] Daelemans D, Afonina E, Nilsson J, Werner G, Kjems J, De Clercq E, Pavlakis GN, Vandamme AM (2002). A synthetic HIV-1 Rev inhibitor interfering with the CRM1-mediated nuclear export. Proc Natl Acad Sci U S A.

[21] Meissner T, Krause E, Vinkemeier U (2004). Ratjadone and leptomycin B block CRM1-dependent nuclear export by identical mechanisms. FEBS Lett.

[22] Newlands ES, Rustin GJ, Brampton MH (1996). Phase I trial of elactocin. Br J Cancer.

[23] Etchin J, Sun Q, Kentsis A, Farmer A, Zhang ZC, Sanda T, Mansour MR, Barcelo C, McCauley D, Kauffman M, Shacham S, Christie AL, Kung AL, Rodig SJ, Chook YM, Look AT (2013). Antileukemic activity of nuclear export inhibitors that spare normal hematopoietic cells. Leukemia.

[24] Lapalombella R, Sun Q, Williams K, Tangeman L, Jha S, Zhong Y, Goettl V, Mahoney E, Berglund C, Gupta S, Farmer A, Mani R, Johnson AJ, Lucas D, Mo X, Daelemans D, Sandanayaka V, Shechter S, McCauley D, Shacham S, Kauffman M, Chook YM, Byrd JC (2012). Selective inhibitors of nuclear export show that CRM1/XPO1 is a target in chronic lymphocytic leukemia. Blood.

[25] Ranganathan P, Yu X, Na C, Santhanam R, Shacham S, Kauffman M, Walker A, Klisovic R, Blum W, Caligiuri M, Croce CM, Marcucci G, Garzon R (2012). Preclinical activity of a novel CRM1 inhibitor in acute myeloid leukemia. Blood.

[26] Azmi AS, Aboukameel A, Bao B, Sarkar FH, Philip PA, Kauffman M, Shacham S, Mohammad RM (2013). Selective inhibitors of nuclear export block pancreatic cancer cell proliferation and reduce tumor growth in mice. Gastroenterology.

[27] Sells SF, Han SS, Muthukkumar S, Maddiwar N, Johnstone R, Boghaert E, Gillis D, Liu G, Nair P, Monnig S, Collini P, Mattson MP, Sukhatme VP, Zimmer SG, Wood DP, McRoberts JW, Shi Y, Rangnekar VM (1997). Expression and function of the leucine zipper protein Par-4 in apoptosis. Mol Cell Biol.

[28] Ahmed MM, Sheldon D, Fruitwala MA, Venkatasubbarao K, Lee EY, Gupta S, Wood C, Mohiuddin M, Strodel WE (2008). Downregulation of PAR-4, a pro-apoptotic gene, in pancreatic tumors harboring K-ras mutation. Int J Cancer.

[29] Tanaka K, Iwamoto S, Gon G, Nohara T, Iwamoto M, Tanigawa N (2000). Expression of survivin and its relationship to loss of apoptosis in breast carcinomas. Clin Cancer Res.

[30] Dohi T, Okada K, Xia F, Wilford CE, Samuel T, Welsh K, Marusawa H, Zou H, Armstrong R, Matsuzawa S, Zou H, Armstrong R, Matsuzawa S, Salvesen GS, Reed JC, Altieri DC (2004). An IAP-IAP complex inhibits apoptosis. J Biol Chem.

[31] Furqan M, Mukhi N, Lee B, Liu D (2013). Dysregulation of JAK-STAT pathway in hematological malignancies and JAK inhibitors for clinical application. Biomarker Res.

[32] Bromberg J, Darnell JE (2000). The role of STATs in transcriptional control and their impact on cellular function. Oncogene.

[33] Rodríguez JA, Span SW, Ferreira CG, Kruyt FA, Giaccone G (2002). CRM1-mediated nuclear export determines the cytoplasmic localization of the antiapoptotic protein Survivin. Exp Cell Res.

[34] Stauber RH, Rabenhorst U, Rekik A, Engels K, Bier C, Knauer SK (2006). Nucleocytoplasmic shuttling and the biological activity of mouse survivin are regulated by an active nuclear export signal. Traffic.

[35] Mau-Soerensen M, Razak ARA, Shields AF, Gabrail NY, Gerecitano JF, Shacham S, Lassen UN, Rashal T, Cooksey J, Landesman Y, Pond G, Oza AM, Kauffman M, Siu LL, Bedard PL, Mahaseth H, Mirza MR, Mahipal A (2014). A first-in-class, first-in-human phase I trial of KPT-330 (selinexor), a selective inhibitor of nuclear export (SINE) in patients (pts) with advanced solid tumors. ASCO Meeting Abstracts.

[36] Xu C, Zhou Q, Wu Y-l (2012). Can EGFR-TKIs be used in first line treatment for advanced non-small cell lung cancer based on selection according to clinical factors? – A literature-based meta-analysis. J Hematol Oncol.

[37] Niu F-Y, Wu Y-L (2014). Novel agents and strategies for overcoming EGFR TKIs resistance. Exp Hematol Oncol.

[38] Sharma SV, Bell DW, Settleman J, Haber DA (2007). Epidermal growth factor receptor mutations in lung cancer. Nat Rev Cancer.

[39] Stewart DJ (2010). Tumor and host factors that may limit efficacy of chemotherapy in non-small cell and small cell lung cancer. Crit Rev Oncol Hematol.

[40] Stommel JM, Marchenko ND, Jimenez GS, Moll UM, Hope TJ, Wahl GM (1999). A leucine-rich nuclear export signal in the p53 tetramerization domain: regulation of subcellular localization and p53 activity by NES masking. EMBO J.

[41] Sun H, Hattori N, Chien W, Sun Q, Sudo M, GL EL, Ding L, Lim SL, Shacham S, Kauffman M, Nakamaki T, Koeffler HP (2014). KPT-330 has antitumour activity against non-small cell lung cancer. Br J Cancer.

[42] Moll UM, Slade N (2004). p63 and p73: roles in development and tumor formation. Mol Cancer Res.

[43] Wang S, Han X, Wang J, Yao J, Shi Y (2014). Antitumor effects of a novel chromosome region maintenance 1 (CRM1) inhibitor on non-small cell lung cancer cells in vitro and in mouse tumor xenografts. PLoS One.

[44] Belldegrun AS, Klatte T, Shuch B, LaRochelle JC, Miller DC, Said JW, Riggs SB, Zomorodian N, Kabbinavar FF, Dekernion JB, Pantuck AJ (2008). Cancer-specific survival outcomes among patients treated during the cytokine era of kidney cancer (1989–2005): a benchmark for emerging targeted cancer therapies. Cancer.

[45] Inoue H, Kauffman M, Shacham S, Landesman Y, Yang J, Evans CP, Weiss RH (2013). CRM1 blockade by selective inhibitors of nuclear export attenuates kidney cancer growth. J Urol.

[46] Inoue H, Hwang SH, Wecksler AT, Hammock BD, Weiss RH (2011). Sorafenib attenuates p21 in kidney cancer cells and augments cell death in combination with DNA-damaging chemotherapy. Cancer Biol Ther.

[47] Jerant AF, Johnson JT, Sheridan CD, Caffrey TJ (2000). Early detection and treatment of skin cancer. Am Fam Physician.

[48] Atefi M, von Euw E, Attar N, Ng C, Chu C, Guo D, Nazarian R, Chmielowski B, Glaspy JA, Comin-Anduix B, Mischel PS, Lo RS, Ribas A (2011). Reversing melanoma cross-resistance to BRAF and MEK inhibitors by co-targeting the AKT/mTOR pathway. PLoS One.

[49] Baldus SE, Schaefer KL, Engers R, Hartleb D, Stoecklein NH, Gabbert HE (2010). Prevalence and heterogeneity of KRAS, BRAF, and PIK3CA mutations in primary colorectal adenocarcinomas and their corresponding metastases. Clin Cancer Res.

[50] Dalle S, Poulalhon N, Thomas L (2011). Vemurafenib in melanoma with BRAF V600E mutation. N Engl J Med.

[51] Davies H, Bignell GR, Cox C, Stephens P, Edkins S, Clegg S, Teague J, Woffendin H, Garnett MJ, Bottomley W, Davis N, Dicks E, Ewing R, Floyd Y, Gray K, Hall S, Hawes R, Hughes J, Kosmidou V, Menzies A, Mould C, Parker A, Stevens C, Watt S, Hooper S, Wilson R, Jayatilake H, Gusterson BA, Cooper C, Shipley J (2002). Mutations of the BRAF gene in human cancer. Nature.

[52] Lee B, Mukhi N, Liu D (2012). Current management and novel agents for malignant melanoma. J Hematol Oncol.

[53] Akinleye A, Furqan M, Mukhi N, Ravella P, Liu D (2013). MEK and the inhibitors: from bench to bedside. J Hematol Oncol.

[54] Huang T, Karsy M, Zhuge J, Zhong M, Liu D (2013). B-Raf and the inhibitors: from bench to bedside. J Hematol Oncol.

[55] Ascierto PA, Schadendorf D, Berking C, Agarwala SS, van Herpen CM, Queirolo P, Blank CU, Hauschild A, Beck JT, St-Pierre A, Niazi F, Wandel S, Peters M, Zubel A, Dummer R (2013). MEK162 for patients with advanced melanoma harbouring NRAS or Val600 BRAF mutations: a non-randomised, open-label phase 2 study. Lancet Oncol.

[56] Chapman PB, Hauschild A, Robert C, Haanen JB, Ascierto P, Larkin J, Dummer R, Garbe C, Testori A, Maio M, Hogg D, Lorigan P, Lebbe C, Jouary T, Schadendorf D, Ribas A, O’Day SJ, Sosman JA, Kirkwood JM, Eggermont AM, Dreno B, Nolop K, Li J, Nelson B, Hou J, Lee RJ, Flaherty KT, McArthur GA (2011). Improved survival with vemurafenib in melanoma with BRAF V600E mutation. N Engl J Med.

[57] Flaherty KT, Robert C, Hersey P, Nathan P, Garbe C, Milhem M, Demidov LV, Hassel JC, Rutkowski P, Mohr P, Dummer R, Trefzer U, Larkin JM, Utikal J, Dreno B, Nyakas M, Middleton MR, Becker JC, Casey M, Sherman LJ, Wu FS, Ouellet D, Martin AM, Patel K, Schadendorf D (2012). Improved survival with MEK inhibition in BRAF-mutated melanoma. N Engl J Med.

[58] Ho AL, Grewal RK, Leboeuf R, Sherman EJ, Pfister DG, Deandreis D, Pentlow KS, Zanzonico PB, Haque S, Gavane S, Ghossein RA, Ricarte-Filho JC, Dominguez JM, Shen R, Tuttle RM, Larson SM, Fagin JA (2013). Selumetinib-enhanced radioiodine uptake in advanced thyroid cancer. N Engl J Med.

[59] Janne PA, Shaw AT, Pereira JR, Jeannin G, Vansteenkiste J, Barrios C, Franke FA, Grinsted L, Zazulina V, Smith P, Crino L (2013). Selumetinib plus docetaxel for KRAS-mutant advanced non-small-cell lung cancer: a randomised, multicentre, placebo-controlled, phase 2 study. Lancet Oncol.

[60] Marks JL, Gong Y, Chitale D, Golas B, McLellan MD, Kasai Y, Ding L, Mardis ER, Wilson RK, Solit D, Levine R, Michel K, Thomas RK, Rusch VW, Ladanyi M, Pao W (2008). Novel MEK1 mutation identified by mutational analysis of epidermal growth factor receptor signaling pathway genes in lung adenocarcinoma. Cancer Res.

[61] Pathria G, Wagner C, Wagner SN (2012). Inhibition of CRM1-mediated nucleocytoplasmic transport: triggering human melanoma cell apoptosis by perturbing multiple cellular pathways. J Invest Dermatol.

[62] Salas Fragomeni RA, Chung HW, Landesman Y, Senapedis W, Saint-Martin JR, Tsao H, Flaherty KT, Shacham S, Kauffman M, Cusack JC (2013). CRM1 and BRAF inhibition synergize and induce tumor regression in BRAF-mutant melanoma. Mol Cancer Ther.

[63] Cagnol S, Chambard JC (2010). ERK and cell death: mechanisms of ERK-induced cell death–apoptosis, autophagy and senescence. FEBS J.

[64] Mirmohammadsadegh A, Mota R, Gustrau A, Hassan M, Nambiar S, Marini A, Bojar H, Tannapfel A, Hengge UR (2007). ERK1/2 is highly phosphorylated in melanoma metastases and protects melanoma cells from cisplatin-mediated apoptosis. J Invest Dermatol.

[65] Kojima K, Kornblau SM, Ruvolo V, Dilip A, Duvvuri S, Davis RE, Zhang M, Wang Z, Coombes KR, Zhang N, Qiu YH, Burks JK, Kantarjian H, Shacham S, Kauffman M, Andreeff M (2013). Prognostic impact and targeting of CRM1 in acute myeloid leukemia. Blood.

[66] Saha M, Qiu L, Chang H (2013). Targeting p53 by small molecules in hematological malignancies. J Hematol Oncol.

[67] Pawar R, Singh Bali OP, Malhotra BK, Lamba G (2014). Recent advances and novel agents for FLT3 mutated AML. Stem Cell Invest.

[68] Cheng K, Sportoletti P, Ito K, Clohessy JG, Teruya-Feldstein J, Kutok JL, Pandolfi PP (2010). The cytoplasmic NPM mutant induces myeloproliferation in a transgenic mouse model. Blood.

[69] Yoon K, Smart RC (2004). C/EBPalpha is a DNA damage-inducible p53-regulated mediator of the G1 checkpoint in keratinocytes. Mol Cell Biol.

[70] Keeshan K, Santilli G, Corradini F, Perrotti D, Calabretta B (2003). Transcription activation function of C/EBPalpha is required for induction of granulocytic differentiation. Blood.

[71] Tenen DG, Hromas R, Licht JD, Zhang DE (1997). Transcription factors, normal myeloid development, and leukemia. Blood.

[72] Mrózek K, Marcucci G, Paschka P, Whitman SP, Bloomfield CD (2007). Clinical relevance of mutations and gene-expression changes in adult acute myeloid leukemia with normal cytogenetics: are we ready for a prognostically prioritized molecular classification?. Blood.

[73] Ikeda H, Kanakura Y, Tamaki T, Kuriu A, Kitayama H, Ishikawa J, Kanayama Y, Yonezawa T, Tarui S, Griffin JD (1991). Expression and functional role of the proto-oncogene c-kit in acute myeloblastic leukemia cells. Blood.

[74] Paschka P, Marcucci G, Ruppert AS, Mrózek K, Chen H, Kittles RA, Vukosavljevic T, Perrotti D, Vardiman JW, Carroll AJ, Kolitz JE, Larson RA, Bloomfield CD, CaLG B (2006). Adverse prognostic significance of KIT mutations in adult acute myeloid leukemia with inv (16) and t (8;21): a Cancer and Leukemia Group B Study. J Clin Oncol.

[75] Kojima K, Konopleva M, McQueen T, O’Brien S, Plunkett W, Andreeff M (2006). Mdm2 inhibitor Nutlin-3a induces p53-mediated apoptosis by transcription-dependent and transcription-independent mechanisms and may overcome Atm-mediated resistance to fludarabine in chronic lymphocytic leukemia. Blood.

[76] Etchin J, Sanda T, Mansour MR, Kentsis A, Montero J, Le BT, Christie AL, McCauley D, Rodig SJ, Kauffman M, Shacham S, Stone R, Letai A, Kung AL, Thomas Look A (2013). KPT-330 inhibitor of CRM1 (XPO1)-mediated nuclear export has selective anti-leukaemic activity in preclinical models of T-cell acute lymphoblastic leukaemia and acute myeloid leukaemia. Br J Haematol.

[77] Pui CH, Evans WE (2006). Treatment of acute lymphoblastic leukemia. N Engl J Med.

[78] Pui CH, Robison LL, Look AT (2008). Acute lymphoblastic leukaemia. Lancet.

[79] Zhao Y, Huang H, Wei G (2013). Novel agents and biomarkers for acute lymphoid leukemia. J Hematol Oncol.

[80] Farnsworth P, Ward D, Reddy V (2012). Persistent complete molecular remission after nilotinib and graft-versus-leukemia effect in an acute lymphoblastic leukemia patient with cytogenetic relapse after allogeneic stem cell transplantation. Exp Hematol Oncol.

[81] Nagafuji K, Miyamoto T, Eto T, Kamimura T, Taniguchi S, Okamura T, Ohtsuka E, Yoshida T, Higuchi M, Yoshimoto G, Fujisaki T, Abe Y, Takamatsu Y, Yokota S, Akashi K, Harada M (2013). Monitoring of minimal residual disease (MRD) is useful to predict prognosis of adult patients with Ph-negative ALL: results of a prospective study (ALL MRD2002 Study). J Hematol Oncol.

[82] Walker CJ, Oaks JJ, Santhanam R, Neviani P, Harb JG, Ferenchak G, Ellis JJ, Landesman Y, Eisfeld AK, Gabrail NY, Smith CL, Caligiuri MA, Hokland P, Roy DC, Reid A, Milojkovic D, Goldman JM, Apperley J, Garzon R, Marcucci G, Shacham S, Kauffman MG, Perrotti D (2013). Preclinical and clinical efficacy of XPO1/CRM1 inhibition by the karyopherin inhibitor KPT-330 in Ph + leukemias. Blood.

[83] Yee KWL, Savona M, Sorensen M, Brown P, Blum WG, DeAngelo DJ, Gutierrez M, Garzon R, Schuh AC, Gabrail NY, Wadleigh M, Lancet JE, Shah BD, Berdeja JG, Wagner-Johnston ND, Flinn I, Rashal T, Kauffman M, Shacham S, Stone RM (2014). A phase 1 dose-escalation study of the oral selective inhibitor of nuclear export (SINE) KPT-330 (selinexor) in patients (pts) with relapsed/refractory acute myeloid leukemia (AML). ASCO Meeting Abstracts.

[84] Akinleye A, Avvaru P, Furqan M, Song Y, Liu D (2013). Phosphatidylinositol 3-kinase (PI3K) inhibitors as cancer therapeutics. J Hematol Oncol.

[85] Akinleye A, Chen Y, Mukhi N, Song Y, Liu D (2013). Ibrutinib and novel BTK inhibitors in clinical development. J Hematol Oncol.

[86] Lu K, Wang X (2012). Therapeutic advancement of chronic lymphocytic leukemia. J Hematol Oncol.

[87] Wu M, Akinleye A, Zhu X (2013). Novel agents for chronic lymphocytic leukemia. J Hematol Oncol.

[88] Cang S, Mukhi N, Wang K, Liu D (2012). Novel CD20 monoclonal antibodies for lymphoma therapy. J Hematol Oncol.

[89] Wang K, Wei G, Liu D (2012). CD19: a biomarker for B cell development, lymphoma diagnosis and therapy. Exp Hematol Oncol.

[90] Han E, Li X-L, Wang C-R, Li T-F, Han S-Y (2013). Chimeric antigen receptor-engineered T cells for cancer immunotherapy: progress and challenges. J Hematol Oncol.

[91] Chen Y, Liu D (2014). Chimeric antigen receptor (CAR)-directed adoptive immunotherapy: a new era in targeted cancer therapy. Stem Cell Invest.

[92] Furman RR, Asgary Z, Mascarenhas JO, Liou HC, Schattner EJ (2000). Modulation of NF-kappa B activity and apoptosis in chronic lymphocytic leukemia B cells. J Immunol.

[93] Reed JC, Pellecchia M (2005). Apoptosis-based therapies for hematologic malignancies. Blood.

[94] Schmidt J, Braggio E, Kortuem KM, Egan JB, Zhu YX, Xin CS, Tiedemann RE, Palmer SE, Garbitt VM, McCauley D, Kauffman M, Shacham S, Chesi M, Bergsagel PL, Stewart AK (2013). Genome-wide studies in multiple myeloma identify XPO1/CRM1 as a critical target validated using the selective nuclear export inhibitor KPT-276. Leukemia.

[95] Grinberg AV, Hu CD, Kerppola TK (2004). Visualization of Myc/Max/Mad family dimers and the competition for dimerization in living cells. Mol Cell Biol.

[96] Chng WJ, Huang GF, Chung TH, Ng SB, Gonzalez-Paz N, Troska-Price T, Mulligan G, Chesi M, Bergsagel PL, Fonseca R (2011). Clinical and biological implications of MYC activation: a common difference between MGUS and newly diagnosed multiple myeloma. Leukemia.

[97] Golomb L, Bublik DR, Wilder S, Nevo R, Kiss V, Grabusic K, Volarevic S, Oren M (2012). Importin 7 and exportin 1 link c-Myc and p53 to regulation of ribosomal biogenesis. Mol Cell.

[98] Delmore JE, Issa GC, Lemieux ME, Rahl PB, Shi J, Jacobs HM, Kastritis E, Gilpatrick T, Paranal RM, Qi J, Hesi M, Schinzel AC, McKeown MR, Heffernan TP, Vakoc CR, Bergsagel PL, Ghobrial IM, Richardson PG, Young RA, Hahn WC, Anderson KC, Kung AL, Bradner JE, Mitsiades CS (2011). BET bromodomain inhibition as a therapeutic strategy to target c-Myc. Cell.

[99] Mochizuki K, Nishiyama A, Jang MK, Dey A, Ghosh A, Tamura T, Natsume H, Yao H, Ozato K (2008). The bromodomain protein Brd4 stimulates G1 gene transcription and promotes progression to S phase. J Biol Chem.

[100] Fang X, Zhou X, Wang X (2013). Clinical development of phosphatidylinositol 3-kinase inhibitors for non-Hodgkin lymphoma. Biomarker Res.

[101] Novero A, Ravella P, Chen Y, Dous G, Liu D (2014). Ibrutinib for B cell malignancies. Exp Hematol Oncol.

[102] Zhao X, Zhang W, Wang L, Zhao W-L (2013). Genetic methylation and lymphoid malignancies: biomarkers of tumor progression and targeted therapy. Biomarker Res.

[103] Fischer K, Cramer P, Busch R, Stilgenbauer S, Bahlo J, Schweighofer CD, Bottcher S, Staib P, Kiehl M, Eckart MJ, Kranz G, Goede V, Elter T, Buhler A, Winkler D, Kneba M, Dohner H, Eichhorst BF, Hallek M, Wendtner CM (2011). Bendamustine combined with rituximab in patients with relapsed and/or refractory chronic lymphocytic leukemia: a multicenter phase II trial of the German Chronic Lymphocytic Leukemia Study Group. J Clin Oncol.

[104] Wierda WG, Padmanabhan S, Chan GW, Gupta IV, Lisby S, Osterborg A (2011). Ofatumumab is active in patients with fludarabine-refractory CLL irrespective of prior rituximab: results from the phase 2 international study. Blood.

[105] Yoshimura M, Ishizawa J, Ruvolo V, Dilip A, Quintás-Cardama A, McDonnell TJ, Neelapu SS, Kwak LW, Shacham S, Kauffman M, Tabe Y, Yokoo M, Kimura S, Andreeff M, Kojima K (2014). Induction of p53-mediated transcription and apoptosis by exportin-1 (XPO1) inhibition in mantle cell lymphoma. Cancer Sci.

[106] Zhang K, Wang M, Tamayo AT, Shacham S, Kauffman M, Lee J, Zhang L, Ou Z, Li C, Sun L, Ford RJ, Pham LV (2013). Novel selective inhibitors of nuclear export CRM1 antagonists for therapy in mantle cell lymphoma. Exp Hematol.

[107] London CA, Bernabe LF, Barnard S, Kisseberth WC, Borgatti A, Henson M, Wilson H, Jensen K, Ito D, Modiano JF, Bear MD, Pennell ML, Saint-Martin JR, McCauley D, Kauffman M, Shacham S (2014). Preclinical evaluation of the novel, orally bioavailable Selective Inhibitor of Nuclear Export (SINE) KPT-335 in spontaneous canine cancer: results of a phase I study. PLoS One.

[108] Gutierrez M, Goy A, Byrd JC, Flynn JM, Sorensen M, Brown P, Gabrail NY, Savona M, Flinn I, Baz RC, Shah BD, Stone RM, Jacobsen E, Kukreti V, Tiedemann RE, Rashal T, Mirza MR, Shacham S, Kauffman M, Kuruvilla J (2014). A phase 1 dose-escalation study of the oral selective inhibitor of nuclear export (SINE) KPT-330 (selinexor) in patients (pts) with heavily pretreated non-Hodgkin lymphoma (NHL). ASCO Meeting Abstracts.

[109] WeiSZ L, Efferth T (2012). Polo-like kinase 1 as target for cancer therapy. Exp Hematol Oncol.

[110] Wang ML, Rule S, Martin P, Goy A, Auer R, Kahl BS, Jurczak W, Advani RH, Romaguera JE, Williams ME, Barrientos JC, Chmielowska E, Radford J, Stilgenbauer S, Dreyling M, Jedrzejczak WW, Johnson P, Spurgeon SE, Li L, Zhang L, Newberry K, Ou Z, Cheng N, Fang B, McGreivy J, Clow F, Buggy JJ, Chang BY, Beaupre DM, Kunkel LA (2013). Targeting BTK with ibrutinib in relapsed or refractory mantle-cell lymphoma. N Engl J Med.

[111] Wiestner A (2013). Targeting B-Cell receptor signaling for anticancer therapy: the Bruton's tyrosine kinase inhibitor ibrutinib induces impressive responses in B-cell malignancies. J Clin Oncol.

[112] Woyach JA, Johnson AJ, Byrd JC (2012). The B-cell receptor signaling pathway as a therapeutic target in CLL. Blood.

[113] Zucca E, Bertoni F (2013). Toward new treatments for mantle-cell lymphoma?. N Engl J Med.

[114] Gerecitano J (2014). SINE (selective inhibitor of nuclear export) - translational science in a new class of anti-cancer agents. J Hematol Oncol.

[115] Gravina G, Tortoreto M, Mancini A, Addis A, Di Cesare E, Lenzi A, Landesman Y, McCauley D, Kauffman M, Shacham S, Zaffaroni N, Festuccia C (2014). XPO1/CRM1-Selective Inhibitors of Nuclear Export (SINE) reduce tumor spreading and improve overall survival in preclinical models of prostate cancer (PCa). J Hematol Oncol.

