# Supplementary figures and images for: Selective inhibitors of nuclear export (SINE)– a novel class of anti-cancer agents
Source: J Hematol Oncol. 2014 Oct 15;7:78. doi: 10.1186/s13045-014-0078-0 (PMC4200201; doi:10.1186/s13045-014-0078-0)

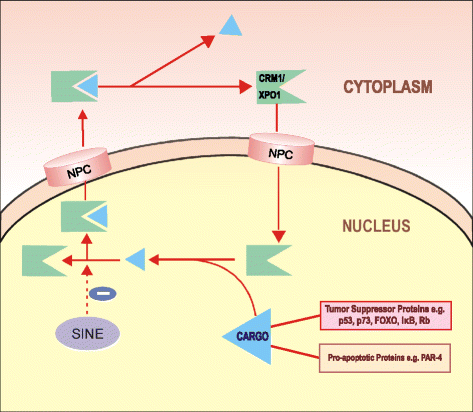

Supplement: Supplementary file 1 — Authors’ original file for figure 1 [file 13045_2014_78_MOESM1_ESM.gif]
